# At home with eating disorders

**DOI:** 10.1186/2050-2974-1-S1-O19

**Published:** 2013-11-14

**Authors:** Elaine Painter, Kirsty Greenwood, Belinda Caldwell, Jeremy Freeman

**Affiliations:** 1 Royal Brisbane & Women's Hospital EDOS, Australia; 2 Butterfly Foundation, Australia; 3 F.E.A.S.T, Australia; 4 ANZAED, Australia

## 

This presentation will discuss the outcomes and evaluations of the organisation and delivery of the 2013 "At home with eating disorders Conference"

## Introduction

With the emergence of evidence based treatments, including Maudsley Family Based Therapy parents and carers play a crucial role in early detection and efficacious treatment. But they do it in the face of significant challenges - lack of information or education, difficulties in navigating the health system, difficulty in finding suitable clinicians, and suffer significant personal and financial burdens in the process.

## Governance

The conference was organised by

- F.E.A.S.T

- The Butterfly Foundation

- Eating Disorder Outreach Service (EDOS)

- ANZAED

## Aim of the conference

This conference aimed to provide access for parents and carers of people with an eating disorder to a range of expert knowledge and skills so they can be better prepared and empowered to take an active role in the treatment of, and recovery from, the eating disorder.

## Measurable outcomes

- Online evaluations pre and post conference

- Attendance,

- Range of Speakers and Workshops

- Budget management

- Successful Sponsorships

Conclusions about the extent to which the conference met its aims, as well as recommendations for future carers conference planning will be made.

This abstract was presented in the **Care in Inpatient and Community Settings** stream of the 2013 ANZAED Conference.

